# Impurity Phases and Hydrogen Decrepitation of Sm_2_TM_17_ Sintered Magnet Production Scrap

**DOI:** 10.3390/nano16040263

**Published:** 2026-02-17

**Authors:** James Griffiths, O. P. Brooks, V. Kozak, H. S. Kitaguchi, A. R. Campbell, A. Lambourne, Richard S. Sheridan

**Affiliations:** 1 School of Metallurgy and Materials, University of Birmingham, Edgbaston, Birmingham B15 2TT, UK; 2 Rolls-Royce Group Plc, Derby DE24 8BJ, UK

**Keywords:** hydrogen, hydrogen decrepitation, magnets, samarium cobalt, magnet recycling

## Abstract

Sm_2_TM_17_ sintered magnets, (where TM = Co, Fe, Cu, Zr), are typically utilised in high temperature magnetic applications due to their magnetic properties being very stable at 200–350 °C. Sm and Co are critical materials and need to be recycled to reduce reliance on virgin material supply chains. This work explored HD processing of Sm_2_TM_17_ sintered magnet production scrap as a potential recycling technique. Sintered magnet scrap was initially analysed compositionally, microstructurally and magnetically to determine issues with magnet quality. Scrap material was then HD processed at 18 bar and 2 bar at temperatures between 25–300 °C. The resultant material was characterised in terms of hydrogen content, particle size, degassing behaviour and unit cell expansion. Production scrap magnets exhibited irregular demagnetisation traces with poor domain wall pinning behaviour. Non-magnetic ZrC inclusions likely prevented cell structure formation locally and hence were poor domain wall pinning sites. Scrap material processed at 18 bar and 2 bar required temperatures of 100 °C to allow for the greatest extent of HD reaction, reaching 0.299 Wt.% and 0.323 Wt.% hydrogen respectively. The HD behaviour of production scrap material was comparable to commercial grade magnets. Therefore, HD is a potentially viable technique for recycling Sm_2_TM_17_ sintered magnet production scrap.

## 1. Introduction

Samarium cobalt 2:17 type sintered magnets (Sm_2_TM_17_) are an intermetallic material where TM typically consists of Co, Fe, Cu and Zr [1,2,3]. These magnets have exceptionally high coercivities (>2000 kA/m) and high Curie temperatures (~850 °C) [4]. These properties, in addition to low thermal coefficients of remanence and coercivity [4], mean that these magnets are the preferred choice for many high temperature magnetic applications such as permanent magnet motors, which are used in the aerospace sector.

Sm_2_TM_17_ sintered magnets rely on a nanoscale cellular structure consisting of three distinct phases to develop their domain wall pinning coercivity mechanism and overall ferromagnetic properties [2,4]. These phases are the Fe-rich rhombohedral phase (2:17R), the Cu-rich hexagonal phase (1:5H) and the Zr-rich rhombohedral phase (1:3R) [5,6,7,8]. The 2:17R phase serves as the main ferromagnetic phase sitting at the centre of the cellular structure, with the 1:5H phase sitting at the cell boundaries [5,6,7,8]. The large magnetocrystalline anisotropy difference between the cell boundary and cell interior gives rise to a domain wall pinning coercivity mechanism. The 1:3R phase acts to form Zr-rich lamellar structures facilitating the diffusion of Cu to the cell boundaries during heat treatment [5,6,7,8].

However, Sm and Co are both critical elements as defined by the European commission and the UK [9,10,11,12,13,14]. This means they are of high economic importance but also great supply risk and that there is growing motivation to recycle these materials to reduce supply chain supply instability [15,16]. It has been proposed that hydrogen decrepitation (HD) could be applied as a recycling technique for end-of-life (EoL) Sm_2_TM_17_ sintered magnets. This process has previously been investigated for ‘short-loop’ magnet-to-magnet recycling with NdFeB sintered magnets [17] and for SmCo_5_ sintered magnets [18].

By exposing Sm_2_TM_17_ sintered magnets to a hydrogen overpressure and a moderate applied temperature (50–150 °C), the material will absorb hydrogen into the 2:17R phase to form an interstitial hydride and expand in volume [15,16,19,20,21,22]. This volume expansion causes intergranular and transgranular cracking and forms a coarse powder. This powder can then be milled, aligned, pressed, sintered and heat treated to form a recycled magnet [15,16,23]. Hydrogen is typically desorbed from HD powders under a vacuum atmosphere at 180–260 °C after HD and/or milling [15,16,20,23,24]. This is because absorbed hydrogen has been linked to ~14% reductions in remanence [20,21] and a 10.4% reduction in (BH)_max_ [16]. Previous investigations have focused on magnet compositions of commercial quality as loose individual sintered magnets and magnets constrained within larger assemblies [15,16,20,21,24].

This article investigates the effects of HD processing on Sm_2_TM_17_ production scrap material, where the magnets did not conform to the required grade of magnetic properties for commercial use. HD could be applied in this scenario as a means to recycle this material back into the production line without having to return to the melting and recasting stage [25]. This could provide a more energy efficient recycling route as the number of processing steps required in short-loop processing is greatly reduced compared to medium-loop (recasting) [25] and long-loop (chemical processing) [26,27,28] recycling techniques.

The reason for these magnets being rejected from commercial use and treated as scrap material may relate to various aspects of the primary production process [4]. The cellular structure that these materials exhibit is responsible for their magnetic properties and hence any deleterious phases introduced during processing will reduce magnetic properties.

This cellular structure forms as a result of a complex set of heat treatments, where a green compact of Sm_2_TM_17_ is sintered at 1190–1220 °C and then homogenised at 1130–1175 °C [29,30,31]. This forms a majority 1:7H metastable phase in the microstructure, which is then quenched to room temperature [29,30,31]. The 1:7H phase is generated as it acts as a precursor phase from which the desired 2:17R will precipitate [32]. The sintered and homogenised compact is then heated to 750–850 °C to isothermally age and form the 2:17R phase and 1:3R phase [32]. During isothermal ageing, 2:7H and 5:19R phases are also precipitated that act as precursors to the 1:5H cell boundary phase [32]. The compact is then cooled at 0.7–1 °C/min to 400 °C from the isothermal ageing temperature. The Zr-rich lamellar structures formed act as diffusion pathways for Cu during this stage, allowing Cu to reach disordered 2:7H and 5:19R phases and form the ordered 1:5H cell boundary phase [32]. Further ageing at 400 °C can be carried out to allow for further diffusion of Cu to cell boundaries and increase the intrinsic coercivity of the magnet.

Compositional irregularities causing the non-ideal phases precipitation could also result in reduced magnetic properties in the sintered magnet [33,34,35]. For example, a very low Sm content may prevent the formation of the 1:7H phase that acts as a direct precursor to the 2:17R phase prior to isothermal ageing [35]. Oxygen and carbon both act as impurities in this system, with oxygen forming the Sm_2_O_3_ phase, which acts to dilute overall magnetic properties and carbon reacting with Zr to form ZrC and prevent the formation of the 1:3R Zr-rich lamellar phase [36,37].

It has been shown that both Sm-rich oxide phases, Zr-rich phases and the grain boundary area of Sm_2_TM_17_ sintered magnets are easy areas of demagnetisation compared to the cellular structure [38,39,40]. Matsuura showed that the grain boundary area and Sm-rich oxide are the first areas to demagnetise in the Sm_2_TM_17_ magnet microstructure when exposed to a reverse magnetic field [38]. The grain boundary area has previously been documented as Cu-lean and hence, in regions near to the grain boundary, the cellular structure of these magnets deteriorates [40]. The lack of cellular structure means that the domain wall pinning coercivity mechanism does not take effect, hence the greater ease of magnetisation reversal in these areas. Sm-rich oxides were documented as non-magnetic inclusions and, therefore, much like the grain boundary areas, were areas where reversal of magnetisation under a reverse applied field occurred more easily [38].

Additionally, 6:23 (Zr_6_Co_23_) and 1:3R Zr-rich primary precipitate phases (not lamellar) have previously been observed in these materials and related to poor processing and heat treatment [39,41]. These phases have been observed on the micron-scale (6:23) and the nanoscale (1:3R) [39]. The presence of either of these phases can lead to areas of the nanostructure lacking 1:5H phase precipitation due to the 6:23 and 1:3R primary precipitate phases acting as defect sinks during isothermal ageing [39]. These undesirable phases are soft magnetic and reduce available domain wall pinning sites.

The applicability of HD processing on materials that may have developed these impurity phases has yet to be explored. Therefore, the impact of non-ideal magnet chemistries and phase structures on the recyclability of these materials using hydrogen processing has yet to be determined.

The aim of this work was to assess the effects of HD processing on non-ideal microstructures/compositions that have developed during primary magnet production. To achieve this, the initial scrap material was first analysed in terms of its composition, nanostructure and magnetic properties to identify why it was not used commercially. The nanostructure of the material was analysed to specifically identify impurity phases and their potential impact on the magnetic properties and HD processing of Sm_2_TM_17_ magnet production scrap.

Magnet production scrap was exposed to HD trials at pressures of 18 bar and 2 bar, at temperatures of 25–300 °C, for a 72 h processing period, and the resulting powders were characterised. Specific emphasis was placed on hydrogen content, particle size/morphology, unit cell expansion and degassing behaviour.

## 2. Methodology

### 2.1. Starting Materials Characterisation

Commercial sintered magnet production scrap was supplied as several large cuboidal blocks, each with a mass of 0.5–5 kg. To provide a fresh reaction surface for decrepitation trials, these blocks were initially cracked using a hydraulic press before HD processing. Commercial-quality reference material of the target magnetic grade was supplied as cuboids with dimensions of 10 × 10 × 11 mm for direct comparison.

Production scrap, reference sintered magnets and HD powders were imaged using a Hitachi 4000+ SEM (Tokyo, Japan) in back-scattered electron mode (BSE), with an accelerating voltage of 15 kV. EDS analysis of the production scrap and reference sintered magnet was conducted using an Oxford Instruments EDS detector (High Wycombe, UK). The microstructure of the Sm_2_TM_17_ scrap material was observed using a FEI Quanta 3D FEG FIB-SEM (Hillsboro, OR, USA) to identify impurity phases and then produce a TEM lamellar of a site of interest. All TEM imaging was conducted perpendicular to the crystallographic c-axis. Production scrap was characterised via a Philips Tecnai F20 transmission electron microscope (TEM) (Amsterdam, The Netherlands) at 200 kV in bright field (BF) imaging mode to examine its cellular structure. The cellular structure was further analysed using a Talos F200X transmission electron microscope (TEM) (Thermo Fisher Scientific, Waltham, MA, USA) at 200 kV, equipped with a high-angle annular dark-field (HAADF) detector with EDS mapping.

XRD phase analysis was conducted on powder samples using a Proto tabletop AXRD (Van Nuys, CA, USA) with a copper lined focus X-ray tube with a nickel kβ absorber (0.02 mm; Kβ = 1.392250 Å) producing Cu Kα radiation (Kα1 = 1.540593 Å, Kα2 = 1.544426 Å). XRD data was processed using CrystalDiffract 7.0 software for the Rietveld refinement of as-received material and HD powders. For simplicity, as only unit cell parameters of the samples analysed were calculated, refinement was completed against the 2:17R crystal structure only.

Both the production scrap and reference material were magnetically characterised using a Magnet Physik EP5 Permagraph (Cologne, Germany) at temperatures of 25, 50, 100 and 200 °C.

Inductively coupled plasma optical emission spectroscopy (ICP-OES) was performed using an Agilent 5110 (Santa Clara, CA, USA) with ~500 mg of sample dissolved in aqua regia. This was used to analyse the compositions of the production scrap and reference material.

### 2.2. Hydrogen Decrepitation Trials

Samples were loaded into an Inconel or stainless steel furnace reactor vessel and evacuated to a 10^−3^ mbar atmosphere. The reactor vessel was then heated, and a hydrogen overpressure was applied. The processing temperatures were 25–300 °C, with a hydrogen pressure of 2 bar or 18 bar for a reaction period of 72 h. The resulting HD powders were weighed and passed through a 1 mm sieve, then the sieved particles were weighed again to calculate the yield of HD powder. All decrepitated samples were stored in a nitrogen-filled MBraun glovebox (Garching, Germany) to limit oxidation of the material. Large-scale HD trials were conducted using a larger tube furnace system sealed with a rubber O-ring, with hydrogen pressure maintained through mass flow controllers set to 2 bar pressure. The flow rate of hydrogen admitted to the system was monitored during hydrogen decrepitation to record hydrogen absorption into the material.

### 2.3. Degassing Trials (Residual Gas Analysis, RGA)

Small samples of HD powder (100 mg) were loaded into a vacuum tube furnace with a MKS HPQ3 residual gas analyser (RGA) attachment (Andover, MA, USA). The vessel was then evacuated to a vacuum pressure of <10^−5^ mbar. The samples were then heated at 5 °C/min to 350 °C, and the RGA unit monitored the hydrogen gas desorbed over time.

### 2.4. Particle Size Analysis

A Malvern Mastersizer 3000 (Malvern, UK) situated inside a nitrogen-filled glovebox was utilised to determine the particle size of the HD powders. To reduce powder agglomerations, the powders were carried through the equipment by nitrogen gas at 4 bar pressure.

### 2.5. Oxygen/Nitrogen/Hydrogen/Carbon Content Analysis

Production scrap and reference sintered magnets were loaded into an Elementrac ONH-p analyser (Eltra, Sarego, Italy) to determine the oxygen and nitrogen content of the alloy using an inert gas fusion technique. Hydrogen content was determined using the same technique with a nitrogen carrier gas and Schuetze reagent.

Samples were also loaded into an Elementrac CS-i Analyser (~500 mg) for carbon analysis. Carbon content was determined by melting in a pure oxygen atmosphere in an induction furnace with temperatures in excess of 2000 °C.

## 3. Results

### 3.1. Scrap Material Characterisation

Table 1 shows the compositional information of the Sm_2_TM_17_ production scrap sample in comparison to that of a reference material. Overall, the compositions were very similar; however, the scrap material had 0.8 Wt.% less Sm content and 740 ppm greater carbon content than the reference material.

**Figure 1:**
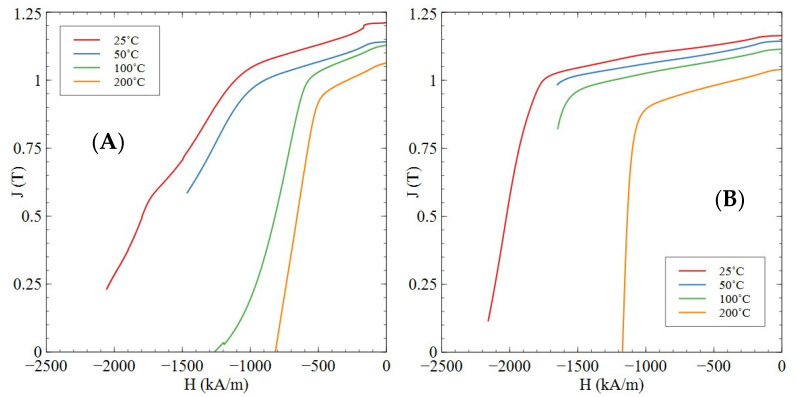
Comparison of demagnetisation behaviour of Sm_2_TM_17_ production scrap (**A**) and the reference material (**B**). Measurements were taken at a range of temperatures from 25 to 200 °C. (**A**) Reprinted from Ref. [23].

Figure 1 shows a comparison of the demagnetisation behaviour of the Sm_2_TM_17_ sintered magnet production scrap and the reference sample of commercial quality.

Measurements of the scrap material at 25 and 50 °C, in addition to the 100 °C measurement for the reference material, did not fully demagnetise and a precise coercivity value could not be provided. During these measurements, the electromagnet used to apply the reverse magnetic field to the sample magnetically saturated before full demagnetisation was achieved. The magnetic properties measured for each sample are shown in Table 2A,B.

It is clear from Table 2A,B that increasing temperature resulted in a reduction in all magnetic properties for both samples, e.g., remanence deceases of 12.4% and 11.2% for the scrap and reference materials, respectively. Figure 1 also showed that the scrap material had a far less square demagnetisation loop compared to the reference material, with multiple fluctuations being seen in the demagnetisation traces presented. The reference material had far more square demagnetisation behaviour, with no noticeable undulations in the demagnetisation traces.

**Figure 2:**
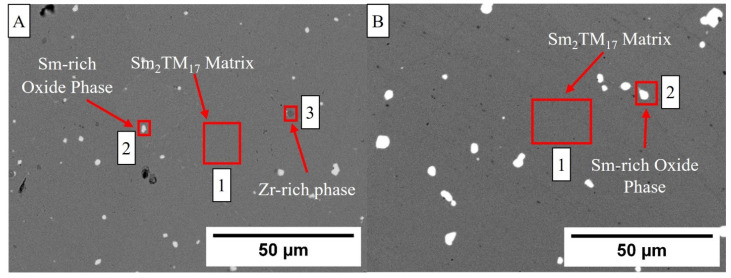
SEM BSE micrographs of the Sm_2_TM_17_ production scrap material (**A**) and the commercial standard reference material (**B**). EDS analysis was carried out at the highlighted areas. (**A**) Reprinted from Ref. [23].

To investigate the reason for this discrepancy in properties, the microstructure and nanostructure of the production scrap material was examined in greater detail. Figure 2 shows a micrograph of the Sm_2_TM_17_ sintered magnet production scrap in comparison to reference material. Both microstructures exhibited a white phase, which was found to be Sm-rich oxide phases (see Table 3 for EDS analysis) and a grey Sm_2_TM_17_ matrix phase. However, there was also a Zr-rich phase located only within the scrap material microstructure, which was recorded as having 68 Wt.% Zr content. This phase was observed as a dark grey feature within the microstructure of the scrap material.

The grain sizes of the production scrap material and reference material have previously been reported as 76 ± 6.5 µm and 176 ± 4.6 µm, respectively [15,23].

**Figure 3:**
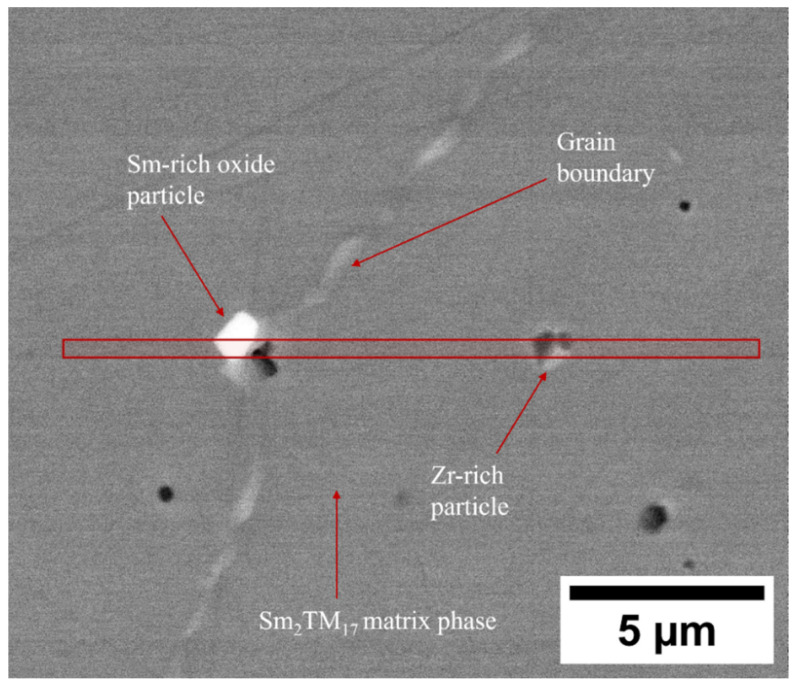
SEM BSE micrograph showing the microstructure of the Sm_2_TM_17_ production scrap. Impurity phases have been highlighted, along with the region from which a TEM lamellar was lifted out (red box).

To further investigate the Sm-rich oxide phases and Zr-rich phases within the production scrap material, a TEM lamellar encompassing these areas and the grain boundary region was prepared using FIB, as shown in Figure 3.

**Figure 4:**
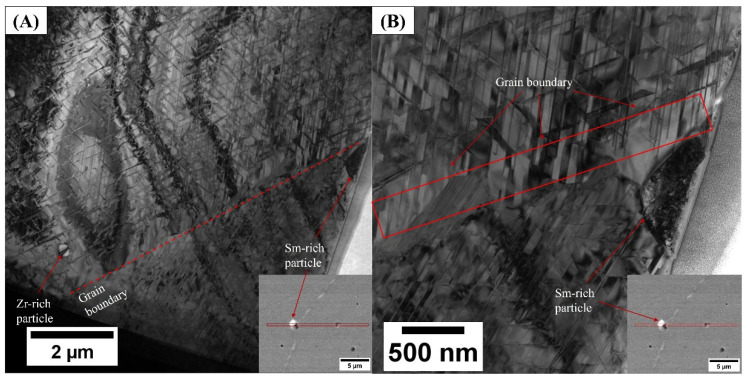
TEM micrographs examining the impurity phases present within Sm_2_TM_17_ sintered magnet production scrap. A low magnification image is presented in image (**A**) and a high magnification image of the grain boundary area is presented in image (**B**).

Figure 4 shows TEM micrographs of the cellular structure of the Sm_2_TM_17_ production scrap. Figure 4A shows a low magnification overview and Figure 4B shows a magnified view of the grain boundary area.

As shown by Figure 4A, the production scrap material displayed a regular diamond-shaped cellular structure across the majority of the area examined. The 2:17R cell interior, 1:5H cell boundary and 1:3R lamellar phases were all observed. These cellular diamond structures were observed to be 434.8 ± 20.7 nm in size.

Additionally, both the Zr-rich and Sm-rich oxide phases were observed as labelled in Figure 4A. The area surrounding both the Zr-rich particle and Sm-rich oxide phase had a noticeably distorted and misshaped cellular structure.

Upon closer examination of Figure 4B, the grain boundary area displayed a very faceted crystal structure. The 1:3R Zr-rich lamellar phases from neighbouring grains did not propagate into the grain boundary area and the typical Sm_2_TM_17_ magnet cellular structure was not observed in the grain boundary.

**Figure 5:**
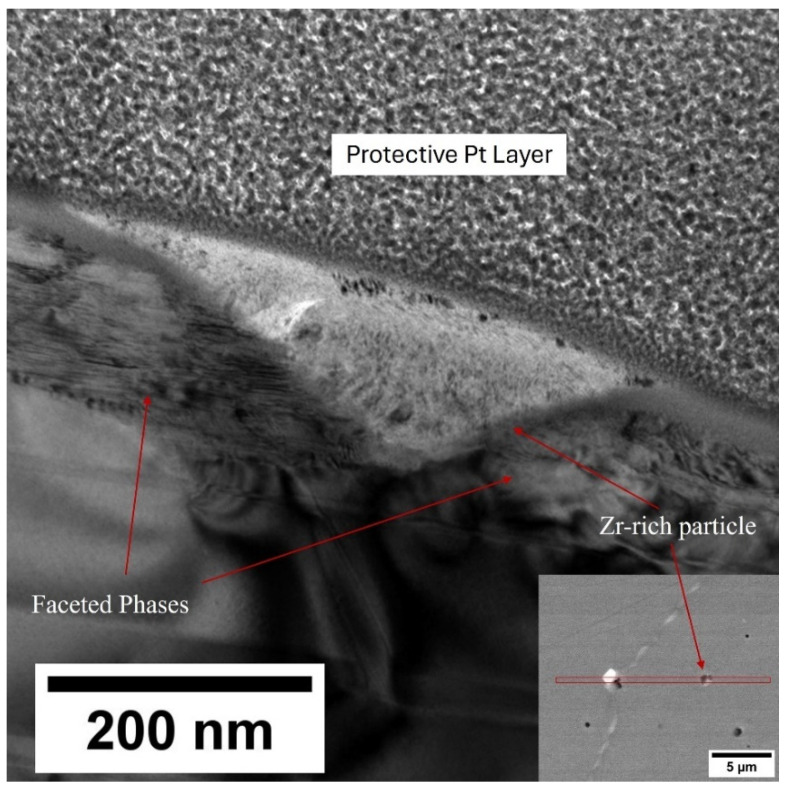
TEM micrograph examining the Zr-rich impurity phase present within the Sm_2_TM_17_ sintered magnet microstructure.

Figure 5 shows a high magnification TEM micrograph of the Zr-rich impurity phase. It was observed that the 1:3R lamellar phase and cellular structure did not develop in close proximity to these phases. Appendix A.1 shows the TEM diffraction pattern of this Zr-rich impurity, with the Zr-rich phase showing an Fm$\bar{3}$m space group with a 4.64 Å lattice parameter. On either side of the Zr-rich phase were crystallographically faceted phases.

### 3.2. EDS Analysis of Impurity Regions

#### 3.2.1. ZrC Impurity Phases

**Figure 6:**
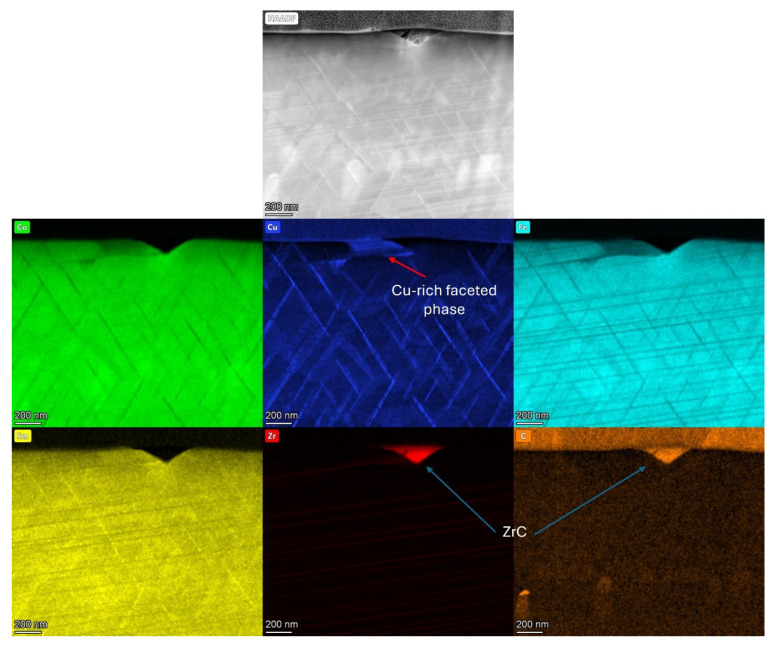
STEM EDS mapping showing a ZrC impurity phase alongside the cellular structure of the production scrap sample.

To identify the composition of the Zr-rich impurity and surrounding phases, EDS mapping was utilised, as shown in Figure 6.

The bulk of the material imaged displayed a cellular structure, with Fe segregating to the cell interior (2:17R), Cu enriching the cell boundary phase (1:5H) and Zr segregating to the lamella phase (1:3R). Co segregated to the 2:17R cell interior and Sm content was greater in the 2:17R cell interior and 1:5H cell boundary phases than in the 1:3R lamellar phase.

The Zr-rich impurity phase was identified as ZrC by EDS mapping, with an extremely high concentration of both elements present in the impurity phase. This phase was entirely depleted of any other alloying elements present in the overall magnet composition. Adjacent to the ZrC phase, significant pooling of Cu was observed. This area of the nanostructure was also observed to be depleted in Co and Fe.

**Figure 7:**
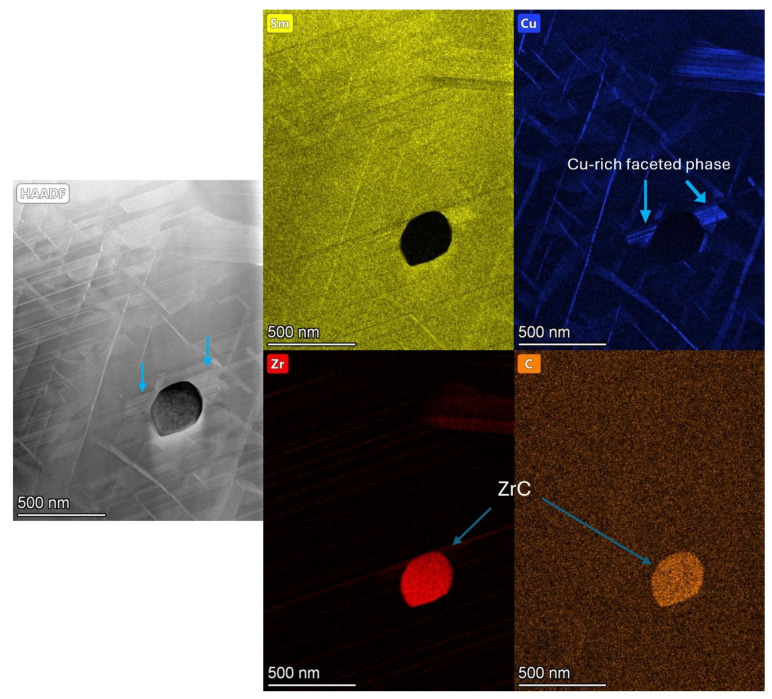
STEM EDS mapping of production scrap material showing ZrC inclusion and a faceted Cu-enriched region local to the ZrC impurity phase.

Further EDS mapping of a larger ZrC inclusion is shown in Figure 7. The ZrC inclusion in this case was circular in morphology and observed to be greatly enriched in Zr and C. A faceted crystal structure was also shown on either side of the impurity phase, which was identified as being Cu-enriched. The region immediately surrounding the ZrC inclusion did not display a uniform cellular structure, with the cell boundary phase being incomplete in these areas. It is important to note that ZrC phases were observed frequently in the sample extracted for TEM analysis and distributed throughout the structure of the material.

#### 3.2.2. Sm-Rich Oxide and Grain Boundary Impurity Phases

EDS mapping was also utilised to investigate the observable compositional differences occurring in the Sm-rich oxide impurity phase and grain boundary regions of the production scrap material.

**Figure 8:**
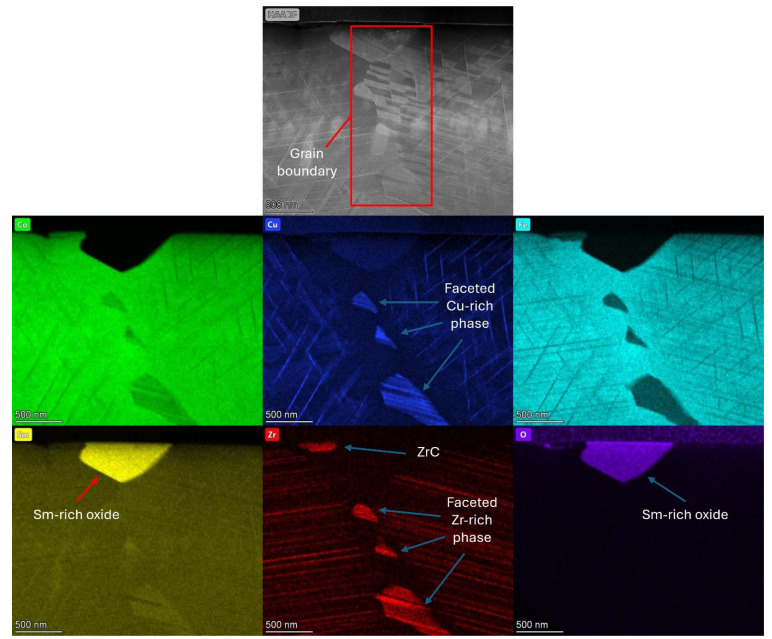
STEM EDS mapping of the Sm-rich oxide phase and grain boundary area.

EDS mapping of both the Sm-rich oxide phase and grain boundary region is shown in Figure 8. The Sm-rich oxide region was depleted of all other alloying elements present within the magnet composition.

The grain boundary region showed a range of faceted phases that were Cu- and Zr-rich. There was clear coherency shown between the Cu- and Zr-rich areas and the grain to the right of the grain boundary.

The observed enrichments and depletions of Cu and Zr appeared to be coupled and therefore likely suggests a correlation between the two. The cellular structure of the Sm_2_TM_17_ production scrap was observed on either side of the grain boundary, but there was a complete loss of this structure in the grain boundary region. It is also of note that the 1:3R lamellar phase changed orientation on either side of the grain boundary, showing a difference in the crystal alignment of the neighbouring grains. This is because the 1:3R phase always has a given orientation relative to the 2:17R phase.

**Figure 9:**
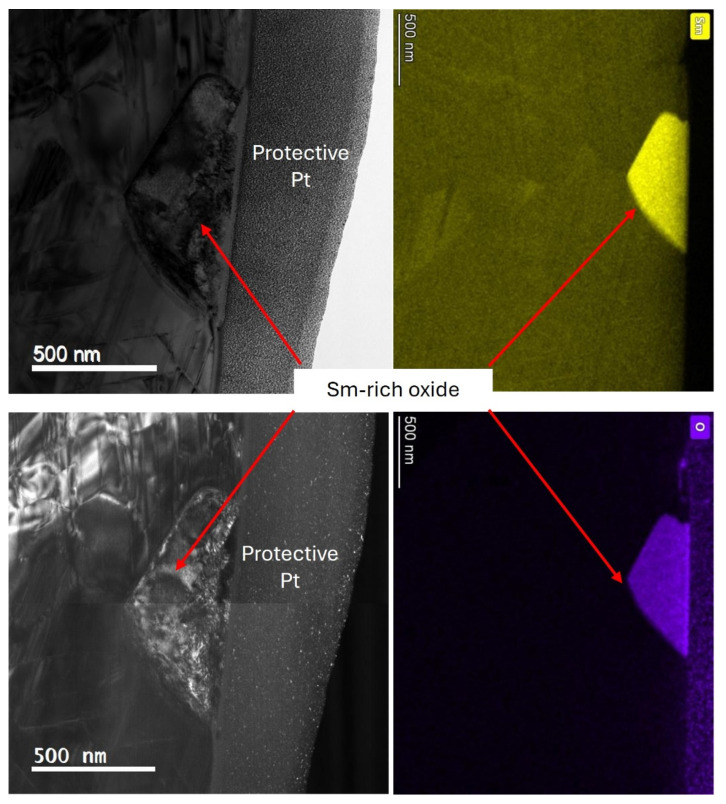
STEM EDS mapping focused on the Sm-rich oxide phase present within the production scrap nanostructure.

To further investigate the Sm-rich oxide phase, EDS mapping was conducted at a greater magnification (see Figure 9). It is clear from Figure 9 that the impurity phase only contained Sm and O, with exceptionally high concentrations of both elements observable in this phase. Furthermore, the TEM diffraction pattern of this phase (as shown in Appendix A.1) indicated that the Sm-rich oxide was monoclinic Sm_2_O_3_-type oxide.

### 3.3. Hydrogen Decrepitation of Production Scrap

**Figure 10:**
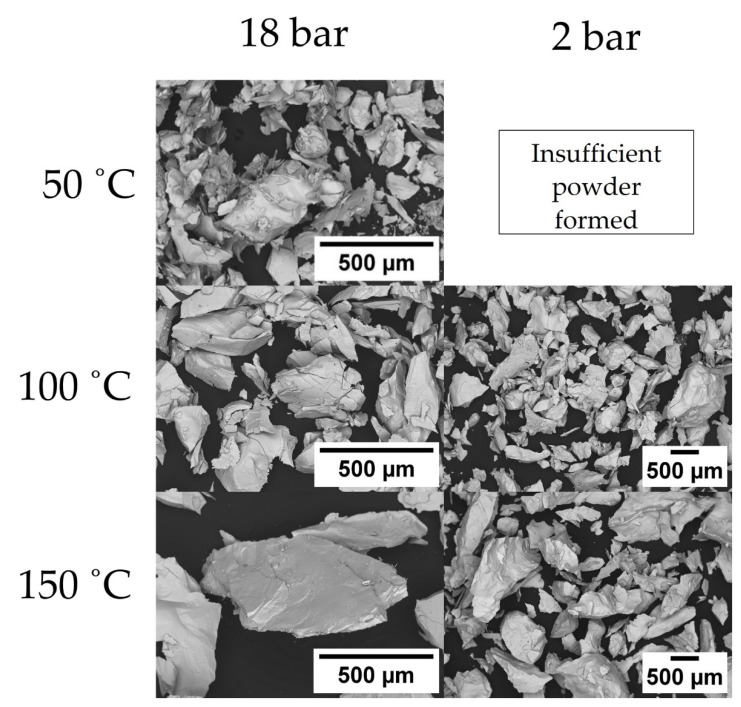
SEM BSE micrographs for HD processing trials of sintered magnet production scrap processed at 18 bar or 2 bar at temperatures of 50, 100 and 150 °C for 72 h.

**Figure 11:**
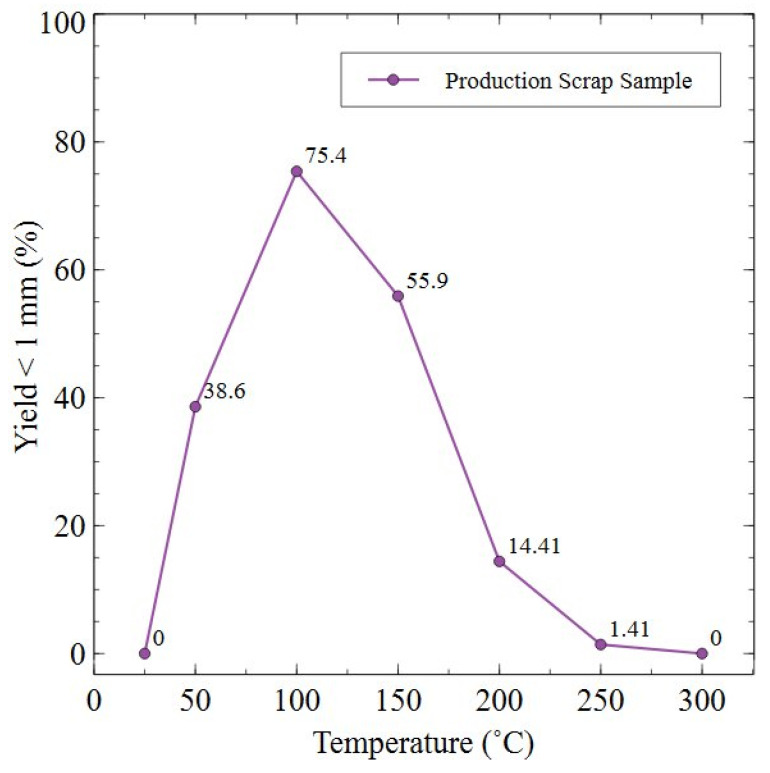
Yield data showing particles with a size < 1 mm for HD trials of sintered magnet production scrap processed at 2 bar at temperatures of 25–300 °C for 72 h.

Figure 10 shows SEM micrographs of HD powders generated at 18 bar and 2 bar across temperatures of 50–150 °C for 72 h, as these trials generated sufficient powder for further analysis. Additional information showing full decrepitation trials at 18 bar at temperatures of 25–150 °C and 2 bar at temperatures of 25–300 °C are shown in Appendix A.2. Additional yield data showing the percentage of particles < 1 mm in size for the 2 bar trials is shown in Figure 11.

As shown by the micrographs in Figure 10, both the 18 bar and 2 bar powders had a flake-like morphology, with increasing temperature resulting in a noticeably coarser powder. Appendix A.2 shows that, in both the 18 bar and 2 bar trials, a temperature of ≥50 °C was required to initiate the HD reaction. The most complete reaction occurred visually at 100 °C for both the 18 and 2 bar trials, as shown in Appendix A.2, with there being no visible bulk solid remaining after the HD reaction.

The yield data presented in Figure 11 supports the finding that, for the 2 bar trials, the HD reaction had progressed further at 100 °C than other temperatures tested. Furthermore, it was evident that at low temperatures of 25 °C or high temperatures of 250/300 °C, the HD reaction was inhibited as powder yield was 0, 1.41 and 0%, respectively. This was also supported by the images in Appendix A.2. These images showed that for each of those respective temperatures, the magnet sample remained either as a bulk solid (25 and 300 °C) or fractured into large chunks (250 °C).

**Figure 12:**
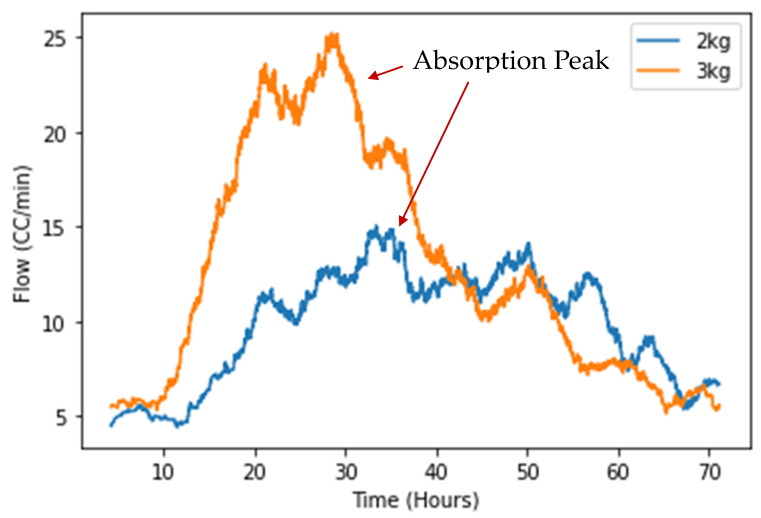
Hydrogen flow rate (moving average) against time for HD reaction of 2 and 3 kg batches of Sm_2_TM_17_ sintered magnet. The processing conditions were 2 bar, 120 °C for 72 h.

Large-scale HD trials at 2 bar, 120 °C for 72 h, were also completed as shown in Appendix A.3, with full decrepitation reactions being observed for 2 and 3 kg batches of scrap material. As hydrogen was admitted into the system by mass flow controller, the hydrogen inlet in each trial was monitored over time and can be used as a method for monitoring when hydrogen was being absorbed by the sample (see Figure 12).

As shown by Figure 12, the reaction for the 2 kg trial initiated after 12 h, with the 3 kg trial reaction initiating after 10 h, as shown by the increased hydrogen flow rate into the system. Following this, there was considerable increase in hydrogen gas flow into the reaction vessel within the first 30 h of both trials. This shows that hydrogen was being absorbed by the magnet production scrap during this period, as the hydrogen content had to be replenished through admission of gas to retain a 2 bar static pressure. Especially in the case of the 3 kg trial, the flow rate into the system rose considerably at the start of the reaction, with the 2 kg trial showing a sustained but lower rate of absorption. By the end of the 72 h trial, hydrogen flow rate into the system for both the 2 and 3 kg trials returned to a value similar to that before the reaction was initiated.

### 3.4. HD Powder Particle Size/Morphology

**Figure 13:**
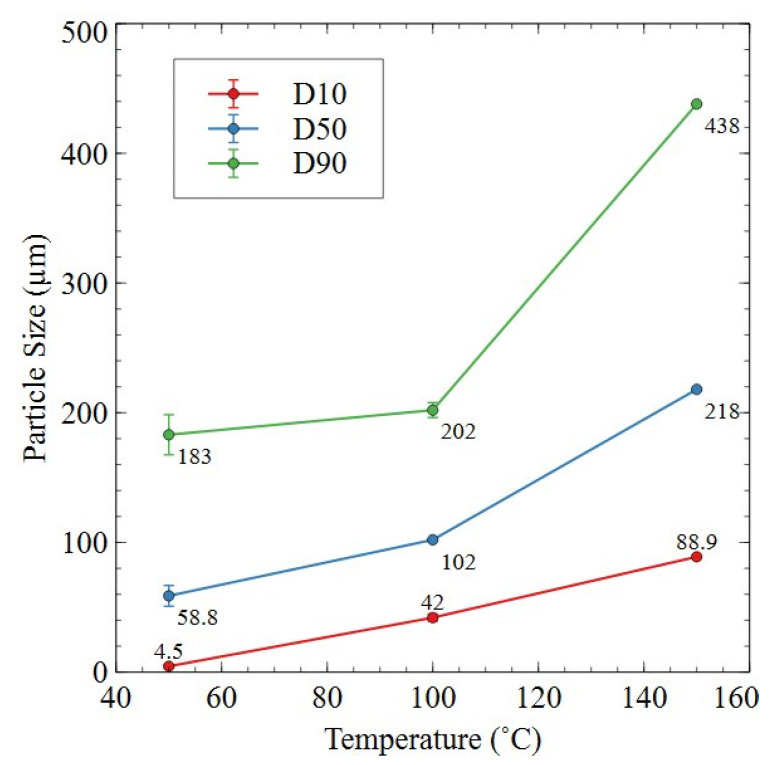
Particle size analysis data for HD powder generated at 18 bar, at 50–150 °C for 72 h. Adapted from Ref. [23].

Figure 13 shows the particle size analysis for Sm_2_TM_17_ sintered magnet production scrap processed at 18 bar at 50–150 °C for 72 h. Particle size distribution is shown through the D10, D50 and D90 values at each processing temperature.

It can be seen from Figure 13 that, with increasing temperature, the particle size generated from processing at 18 bar increased across D10, D50 and D90. The increase in particle size was moderate between 50 and 100 °C measurements, e.g., D90 increased from 183 to 202 µm, but the increase from 100 to 150 °C was far more pronounced. In this case, the D90 increased in size by 117%, with the D50 increasing by 114% and D10 increasing by 112%, showing that the entire particle size distribution shifted to dramatically larger sizes when processing at 150 °C when compared to 100 °C.

**Figure 14:**
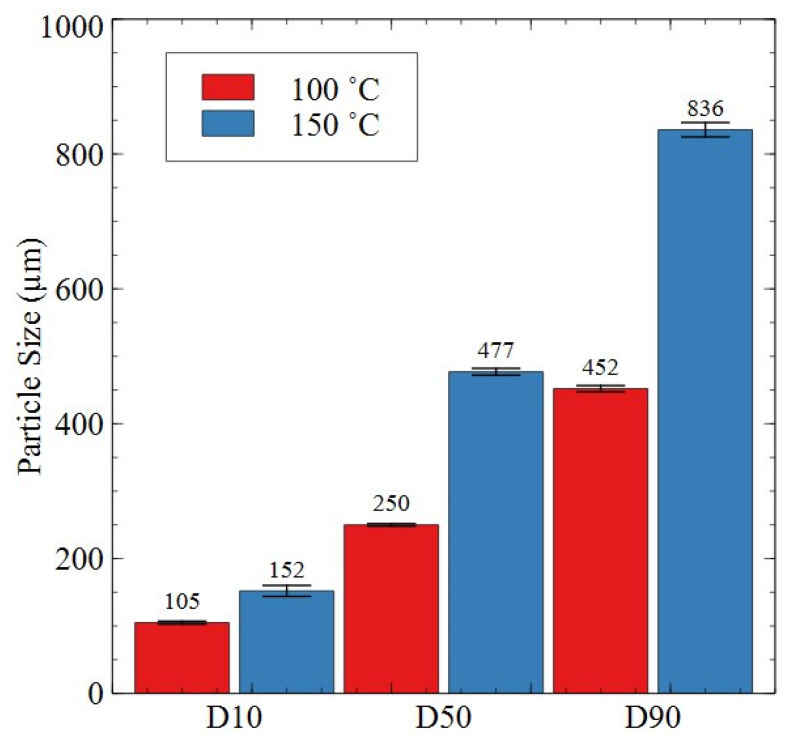
Particle size analysis data for HD powder generated at 2 bar, 100 and 150 °C for 72 h. Adapted from Ref [23].

Figure 14 shows the particle size analysis results for samples processed at 2 bar, 100 or 150 °C, for 72 h.

As shown by Figure 14, the particle size increased across the D10, D50 and D90 percentiles with increasing temperature from 100 to 150 °C. The increase was very prominent, with increases in particle size of 45%, 91% and 85% observed for D10, D50 and D90, respectively.

### 3.5. HD Powder Hydrogen Content Analysis

**Figure 15:**
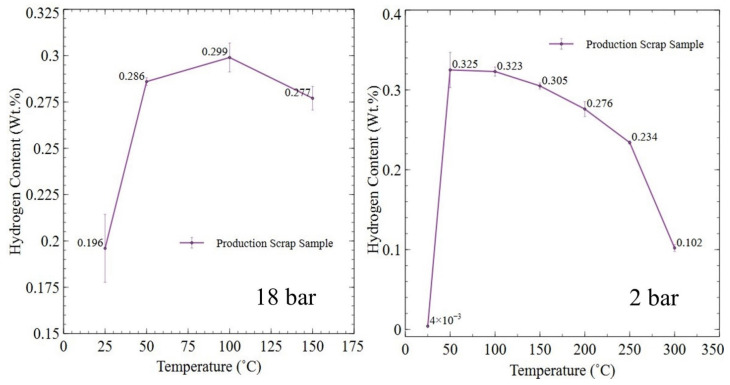
Hydrogen content of production scrap processed at 18 bar or 2 bar, at a variety of temperatures, for 72 h.

Figure 15 shows the hydrogen content of Sm_2_TM_17_ sintered magnet production scrap after HD processing at 18 bar (25–150 °C) and 2 bar (25–300 °C) for 72 h.

For the 18 bar trials, the hydrogen content absorbed due to HD processing reached a peak value at 100 °C, reaching 0.299 Wt.%, with temperatures lower or higher than this both resulting in a reduction in hydrogen content. By far the lowest amount of hydrogen was absorbed at 25 °C, where an uptake of only 0.196 Wt.% was recorded.

The 2 bar trials showed that, at room temperature, there was effectively no hydrogen uptake, with 0.004 Wt.% hydrogen recorded. However, when the temperature was increased to 50 °C, the hydrogen content increased to 0.325 Wt.% and then gradually decreased as temperature was increased to 250 °C, where hydrogen content dropped to 0.234 Wt.%. At 300 °C, there was a sharp reduction in hydrogen content, reaching just 0.102 Wt.%. Overall, when processed at 2 bar, hydrogen content decreased at applied temperatures > 50 °C.

**Figure 16:**
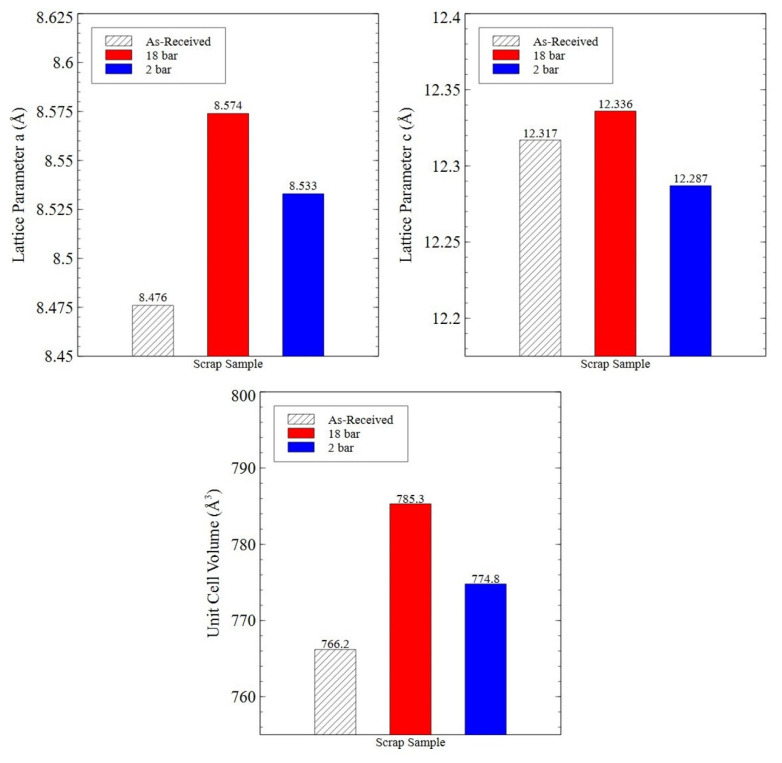
Unit cell lattice parameters of the 2:17R phase within as-received Sm_2_TM_17_ production scrap and HD powder generated at 18 bar and 2 bar, 100 °C for 72 h.

Figure 16 shows the unit cell measurements of the 2:17R phase present in Sm_2_TM_17_ sintered magnet production scrap and HD powder processed at 18 bar or 2 bar, 100 °C for 72 h.

As shown in Figure 16, when HD processed regardless of the hydrogen pressure applied, the 2:17R phase within the scrap material increased in unit cell volume. This increase was more pronounced when the material was processed at 18 bar hydrogen pressure, with an expansion of 19.1 Å^3^ measured, compared to an 8.6 Å^3^ expansion observed in material processed at 2 bar. Both 18 bar and 2 bar HD trials showed that the ‘a’ lattice parameter increased in length due to hydrogen uptake. However, the ‘c’ lattice parameter was observed to increase in material processed at 18 bar but decreased for material processed at 2 bar.

**Figure 17:**
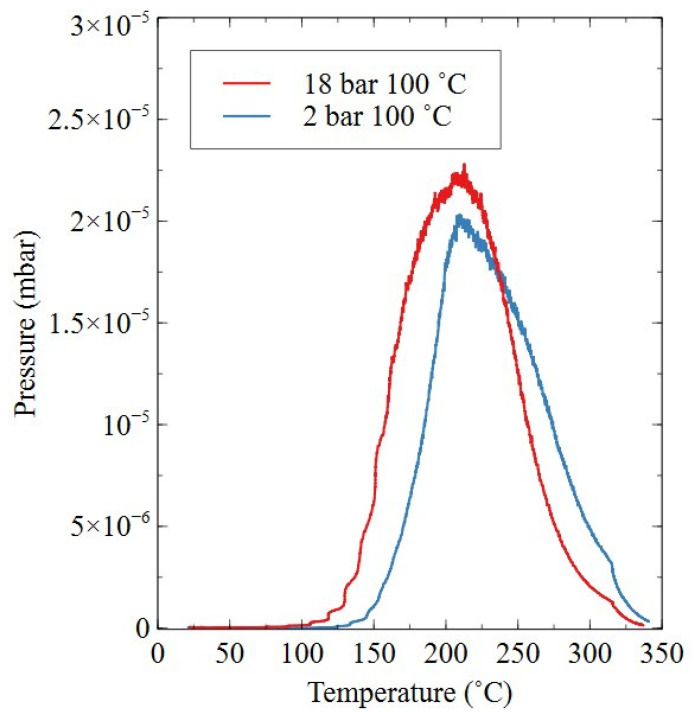
Residual hydrogen gas analysis of Sm_2_TM_17_ production scrap HD powders that were reacted at 18 bar and 2 bar, 100 °C for 72 h.

Figure 17 shows the residual gas analysis of HD powder processed at 18 bar and 2 bar, both at 100 °C, for a processing period of 72 h. The analysis showed that the material processed at 18 bar began to degas at 100 °C, whereas the 2 bar processed powder began to degas at 125 °C. Both samples reached peak hydrogen desorption at 210 °C and degassing was fully complete at 350 °C. Both samples also released hydrogen gas in one degassing step, with no other peaks visible during the analysis.

## 4. Discussion

In this study, Sm_2_TM_17_ sintered magnet production scrap was initially investigated to ascertain why it may not have been of suitable quality to form a commercially viable product. These magnets were then subjected to HD trials at a range of pressures (18 and 2 bar) and temperatures of 25–300 °C for 72 h, and the resultant powders were characterised in terms of hydrogen content, powder size and morphology in addition to degassing behaviour.

Initial compositional analysis comparing the Sm_2_TM_17_ sintered magnet production scrap to the reference target material showed that the scrap material had a lower Sm content by 0.8 Wt.%, higher Co content by 0.4 Wt.%, and 0.4 Wt.% greater Fe compared to the reference material, as shown in Table 1. The Cu and Zr contents of both magnets were very similar and so was the content of nitrogen and oxygen. The major difference between the two, in terms of impurity content, was observed in carbon content. The carbon content of the scrap material was 740 ppm greater in the production scrap sample compared to the reference sample.

It is shown through the demagnetisation analysis in Figure 1 that production scrap material did exhibit ferromagnetic properties consistent with that typically shown by Sm_2_TM_17_ sintered magnets [4,15,21]. However, there were some irregularities within the squareness of the demagnetisation curve shape, which may be linked to the presence of undesirable phases in the microstructure. The magnetic properties of the production scrap material were also very difficult to measure at room temperature as the large reverse magnetic fields often cracked the sintered magnet during measurement. This may be another explanation for undulations in the demagnetisation curve presented.

The TEM analysis presented in Figure 4, Figure 5, Figure 6, Figure 7 and Figure 8 all showed that the production scrap material did display the cellular structure associated with Sm_2_TM_17_ [1,2,3,6,7,8]. This consisted of a clear 2:17R cell interior phase (enriched in Fe), a 1:5H cell boundary phase (enriched in Cu) and a 1:3R lamellar phase (enriched in Zr), comparable to previous Sm_2_TM_17_ sintered magnets subject to HD processing [16,20,21,23]. However, impurity phases were noted in the TEM analysis presented in Figure 4, Figure 5, Figure 6, Figure 7, Figure 8 and Figure 9, which may explain the irregular demagnetisation behaviour of the production scrap material in comparison to the commercial standard reference.

As shown in Figure 2 and Figure 3, and the EDS analysis presented in Table 3, the microstructure of the production scrap material displayed a Zr-rich phase that was not present in the reference sintered magnet. The TEM imaging presented in Figure 5, the diffraction pattern shown in Appendix A.1 and the EDS mapping shown in Figure 6 and Figure 7 all show conclusive evidence that the Zr-rich phase was ZrC. The diffraction pattern shown in Appendix A.1 for the ZrC phase imaged in Figure 5 confirmed a 4.64 Å lattice parameter, with an NaCl-type FCC structure. This is in agreement with previous work by Tian, who identified the ZrC phase within Sm_2_TM_17_ sintered magnets with a lattice parameter of 4.71 Å, determined using X-ray diffraction [36].

Figure 6 and Figure 7 further confirmed the identity of the ZrC with EDS mapping, showing not only a high concentration of Zr in this phase, but C as well. The areas surrounding this phase did not display the cellular structure typical of Sm_2_TM_17_ sintered magnets. A faceted phase with high Cu enrichment formed in close proximity to the ZrC inclusions, as shown in Figure 6 and Figure 7.

As a result of C bonding locally to Zr and forming ZrC, these areas of the microstructure may have seen a reduction in the 1:3R Zr-rich lamellar phase. Consequently, this may have impacted the diffusion of Cu to the 1:5H cell boundary phase during heat treatment [36]. The faceted Cu-rich areas local to the ZrC inclusions may be a result of Cu that was unable to reach the 1:5H phase pooling due to a lack of diffusion pathways, as shown in Figure 6 and Figure 7. An improperly formed cell boundary phase may then impact the domain wall pinning coercivity mechanism of the material due to a lack of magnetocrystalline anisotropy difference between the cell interior and cell boundary [36]. This may provide an explanation for why the demagnetisation behaviour of the production scrap exhibited undulations in its demagnetisation curves measured at 25 and 50 °C. These areas likely indicate that different domain wall pinning mechanisms are at play due to the formation of deleterious phases such as ZrC.

Furthermore, another contributing factor to the irregular demagnetisation behaviour that compliments this point is that the Sm content within the production scrap sample was lower than that of the of the reference sample, being measured at 23.8 and 24.6 Wt.%, respectively, as shown in Table 1. This Sm content is on the lower end of what would be required to enter the 1:7H phase field during homogenisation, with a minimum of 10.5 at.% Sm typically being required [33,34,35]. This relatively low Sm content, combined with the lower effective Zr content due to carbon contamination, could have shifted the ideal temperature required for solution treatment and hence resulted in poor 1:7H phase formation [33,34,35].

Further, the Sm-rich oxide phase identified in Figure 2A and the EDS analysis presented in Table 3 has been shown to demagnetise more readily than the cellular structure when exposed to a reverse magnetic field [38]. This is because it is a non-magnetic inclusion within the microstructure [38]. The nature of the oxide phase was confirmed via EDS analysis, as presented in Figure 8 and Figure 9, with significant segregation of Sm and O observed in these areas. Furthermore, the diffraction pattern shown in Appendix A.1 indicated that the oxide was monoclinic Sm_2_O_3_, which is the most commonly observed oxide in Sm_2_TM_17_ sintered magnets [20,21,42]. However, as the Sm_2_O_3_ was present in both the scrap and reference material microstructures, it is unlikely to be a main cause for the differences in magnetic behaviour observed in Figure 1A,B.

Furthermore, the grain size of the sintered magnet production scrap was 76 ± 6.5 µm compared to 176 ± 4.6 µm for the reference sample [15,23]. Therefore, there were a greater number of grain boundaries within the production scrap material than the reference material. Grain boundaries have been documented as areas that do not form the cellular structure associated with Sm_2_TM_17_ sintered magnets [38,40], as was observed in this work in Figure 4 and Figure 8. The grain boundaries of Sm_2_TM_17_ sintered magnets have been shown to demagnetise at lower reverse applied field magnitudes than the cellular structure and their prevalence influences demagnetisation behaviour [38,40]. EDS mapping of the grain boundary showed clear enrichments and depletions of Cu and Zr coupled with one another, potentially suggesting a correlation between the two (see Figure 8). The areas where these faceted Cu- and Zr-rich phases were observed were also depleted in Co and Fe. Therefore, as the production scrap material had a greater proportion of grain boundary phases, it is possible that this also partly accounts for the lower squareness of demagnetisation behaviour observed for this material compared to the reference sample.

It is possible that, based upon all the aforementioned evidence, the lack of squareness in the demagnetisation section of the hysteresis loop for the Sm_2_TM_17_ sintered magnet production scrap was due to mixed coercivity mechanisms. Typically, a domain wall pinning coercivity mechanism is utilised in Sm_2_TM_17_, but, in close proximity to the faceted grain boundary phases and ZrC or Sm_2_O_3_ impurity phases, coercivity may have switched to nucleation control. Due to the lack of domain wall pinning sites in these areas, reverse domains may have grown rapidly, hence leading to the undulations in the demagnetisation curves shown in Figure 1A.

HD trials completed in this study showed that, for both the 18 and 2 bar trials, an activation temperature of ≥50 °C was required in order for powder to be generated from production scrap, as shown in Figure 10 and Figure 11 and Appendix A.2. This matches well with previous discussion by Schönfeldt and Griffiths, who proposed that in order for Sm_2_TM_17_ sintered magnets to fully react during hydrogen decrepitation, temperatures of >120 °C or of 50–150 °C were required [15,21]. Griffiths also showed that, for low pressures of 2 bar, thermal activation was essential for a reaction to take place. However, at high pressures of 18 bar, some samples could undergo partial or even full decrepitation reactions at room temperature [15]. Interestingly, previous work by Zakotnik showed that the Sm_2_TM_17_ sintered magnets in their study decrepitated under 10 bar pressure at room temperature conditions for 48 h [20]. The composition studied by Griffiths, which decrepitated fully at room temperature and 18 bar pressure, was the same as the reference sample shown in Table 1 in this work, with the sample in the study by Zakotnik having a composition of Sm-51.8Co-14.9Fe-5.5Cu-2.2Zr Wt.% [20]. Griffiths posited that, even for compositionally similar samples, HD behaviour can be very different due to different cell sizes and therefore different quantities of the main hydrogen-absorbing 2:17R phase [15]. The cellular structure of the Sm_2_TM_17_ production scrap was 434.8 ± 20.7 nm, which was much larger than the cell sizes of Sm_2_TM_17_ sintered magnets previously exposed to HD by Griffiths (66–232 nm) [15]. Despite this large cell interior size, the production scrap material did not decrepitate at 25 °C at 18 bar pressure when processed for 72 h. Previously, a larger fraction of the 2:17R phase had been associated with the ability of Sm_2_TM_17_ sintered magnets to decrepitate at room temperature [15], but this data suggest this may not be the only deciding factor.

At 100 °C, the HD reaction resulted in complete powder formation for both the 18 bar and 2 bar trials, as shown in Figure 10 and Appendix A.2. This was also supported by the high yield value of 75.4% of particles < 1 mm, as shown in Figure 11. Additionally, in bulk trials that generated 5 kg of HD powder, 2 bar pressure at 120 °C for 72 h was sufficient for large quantities of scrap to absorb hydrogen and fully fragment into powder, as shown in Figure 12 and Appendix A.3. After 10–12 h, both the 2 kg and 3 kg trials began to absorb hydrogen, with absorption complete after 72 h of processing. This scale of HD processing of sintered Sm_2_TM_17_ magnets has, to the authors’ knowledge, not been attempted before and shows that HD processing of this material is likely scalable to larger commercial capacities.

When HD processed at 100 °C and 18 bar pressure, the production scrap absorbed the greatest amount of hydrogen when compared to all other temperatures tested, reaching 0.299 Wt.% (see Figure 15). A similar pattern was seen in the 2 bar powders with 0.323 Wt.% hydrogen being absorbed, with only the 50 °C trial absorbing slightly more hydrogen, as shown in Figure 15. Despite absorbing more hydrogen at 50 °C, the extent of powder produced was lower for material HD processed at 2 bar at 50 °C. This was likely due to slower reaction kinetics leading to a slower diffusion of hydrogen through the sample, resulting in slower differential volume expansion and cracking.

The need for thermal activation, and the effects of increased temperature on hydrogen diffusion into interstitial hydride-forming intermetallics, has been well documented by Coey and matches the observed phenomena shown in this work [22]. Coey shows that increased temperature at the surface of the intermetallic material facilitates activation of the surface region and allows hydrogen ingress thereafter.

Coey also showed that, with increased temperature, the plateau pressure at which an interstitial rich hydride forms within an intermetallic material gradually increases. This was shown to restrict the ability of hydrogen to be absorbed if a static overpressure and temperature are maintained [22]. This matched well with the observations of hydrogen content for 18 bar and 2 bar trials seen in Figure 15, as, with temperatures > 100 °C, hydrogen content steadily began to decrease. For example, 2 bar trials at 300 °C retained 68% less hydrogen than at 100 °C. This pattern was also observed for commercial grade samples in prior work by Griffiths [15].

An additional supporting point of evidence for reduced hydrogen content was related to the degassing behaviour shown in Figure 17. It was shown that the degassing reaction for samples processed at 18 bar and 2 bar began at 100–125 °C, with maximum hydrogen desorption occurring at 210 °C. This overlaps with the HD processing temperatures applied in the 18 bar and 2 bar trials, indicating that, during trials conducted at ≥150 °C, there were likely competing hydrogen absorption and desorption reactions. This degassing temperature range was similar to that shown by Zakotnik, who showed a temperature of at least 200 °C was required to remove hydrogen from the samples [20,24]. It should be noted that degassing in one of Zakotnik’s studies was completed under an argon atmosphere and not a vacuum, as would be applied industrially. This temperature range and degassing behaviour also matches well with previous discussion by Griffiths on commercial grade samples [15]. It has previously been discussed that hydrogen-induced lattice strain may be responsible for a reduction in remanence and (BH)_max_ within HD powders [16,20,21]. This has been attributed to hydrogen-induced lattice strain disrupting magnetic dipole coupling and reducing the overall magnetocrystalline anisotropy of the material [16]. However, utilising a degassing process, as shown in Figure 17, has been shown to allow for complete restoration of magnetic properties, preventing detrimental effects on the performance of recycled sintered magnets manufactured from HD powders [20].

It was also shown by the particle size analysis results in Figure 13 and Figure 14 that applied pressure and temperature during HD trials can heavily influence the particle size of HD powders. For example, 18 bar trials conducted at 100 °C had a D90 of 202 µm, but, when processed at 150 °C, the D90 had risen to 438 µm. Comparatively, 2 bar trials conducted at 100 °C had a D90 of 452 µm, whereas the sample processed at 150 °C had a D90 of 836 µm. Therefore, an increase in temperature resulted in a coarser powder, with an increase in pressure for equivalent temperature conditions resulting in a finer powder. This result supports previous work on commercial grade samples where the same phenomenon was observed by Griffiths [15].

Further pressure-dependent particle size behaviour was noted by Griffiths, who showed that, under otherwise equivalent HD conditions, powder generated at pressures of 3, 4, 6, 8 and 10 bar showed a gradual reduction in particle size with increasing applied pressure [16]. The reason for this change was likely due to differing hydrogen absorption content and kinetics resulting in differing volume expansion, leading to intergranular and transgranular cracking. By increasing hydrogen pressure, it is clear from the data presented in Figure 13 and Figure 14 and the prior literature that HD powder particle size decreases [15,16,23]. This may be beneficial for further downstream processing, potentially reducing the intensity of milling required to reach particle size distributions of ~2–10 µm for recycled magnet manufacture. However, operating at higher pressures, e.g., 18 bar compared to 2 bar, as in this work, will increase safety concerns and cost implications for reactor pressure vessels on an industrial scale. Therefore, despite the greater particle size reduction afforded by increasing hydrogen pressure, HD processing at lower pressures is likely to be the more industrially viable option.

Additionally, Figure 16 shows that, after HD processing, the 2:17R unit cell expanded by 0.67% in the basal plane, contracted 0.24% on the c-axis and overall increased in volume by 1.12% when processed at 2 bar. This was similar to the expansion noted by Schönfeldt and Griffiths, who noted expansions of the 2:17R unit cell ranging from 1–1.87% depending on the overall hydrogen absorption for fully hydrogen-saturated samples [16,21]. However, the production scrap processed at 18 bar showed a far greater unit cell volume expansion of 2.49% relative to the as-received production scrap. Additionally, the unit cell parameters showed growth in both the ‘a’ and ‘c’ lattice parameters. This differed compared to prior literature, where lattice parameter ‘a’ usually increases in length, but lattice parameter ‘c’ typically decreases in length [16,21]. The greater extent of expansion was attributed to the greater applied pressure compared to prior HD studies. Greater lattice expansion may also explain the far smaller powder particle size generated by processing at higher hydrogen pressures (see Figure 13 and Figure 14 and the prior literature by Griffiths) [15,16,23].

Overall, this research investigated whether HD processing is a viable processing technique to recycle magnets not conforming to commercial quality standard, in this case Sm_2_TM_17_ sintered magnet production scrap. The reason material was classified as scrap was attributed to its poor demagnetisation behaviour, with a very poor demagnetisation trace squareness compared to the reference material. Sm-rich oxide phases, Zr-rich precipitates and the grain boundary area of the scrap material were examined to investigate the potential causes for this irregular cellular demagnetisation behaviour. The Zr-rich phases were identified as ZrC inclusions and, in regions local to this phase, the cellular structure of the production scrap material was diminished. This likely caused poor domain wall pinning and, as these inclusions are non-magnetic, likely acted as easy areas for demagnetisation. Sm-rich oxide phases were noted as other non-magnetic (weakly paramagnetic) areas lacking cellular structure. However, as these phases were present in both the scrap and reference material, the impact of this phase on differences in demagnetisation behaviour was likely minimal. The grain size of the scrap material was 100 µm greater than the reference material and thus the scrap had a much greater proportion of grain boundary phases. This area was also lacking in cellular structure and therefore may have also contributed to the different demagnetisation behaviour observed between the samples.

This research has shown that Sm_2_TM_17_ sintered magnet production scrap can be HD processed in the same way as sintered magnets of a commercial standard. The amount of hydrogen absorbed, unit cell expansion, degassing behaviour and the relationship between applied pressure and particle size all matched well with previous discussion presented in the literature on commercial grade samples. However, though the production scrap material reacts under the same HD processing conditions as commercial material, any impurity phases present within the feedstock material will carry over to recycled magnets manufactured from it. A potential avenue of future research is to examine ways in which the effects of these impurity phases can be mitigated, e.g., the blending in of Zr-rich Sm_2_TM_17_ sintered magnet compositions to compensate for ZrC formation. This may allow for the 1:3R lamellar phase to develop in the blended recycled material, increasing Cu diffusion to the 1:5H boundary phase and improving domain wall pinning behaviour. For instances where impurity content is exceptionally high, HD powders could also serve as a feedstock for chemical processes that allow for recovery of Sm and Co [26,27,28].

Prior investigations by the authors have shown that HD processed magnet production scrap can be degassed, milled, magnetically aligned, pressed, sintered and heat treated into fully dense recycled magnets (8.32 g/cm^3^) [23]. Additionally, Sm-hydride additions of 2–3 Wt.% were shown to be able to compensate for Sm sublimation and oxidation during the recycling process [23]. However, organic surfactants remaining after milling caused even greater precipitation of ZrC inclusions and resulted in recycled magnets with exceptionally low magnetic properties [23].

Therefore, this research suggests that HD processing of Sm_2_TM_17_ sintered magnets using the parameters previously applied to commercial grade magnets can also be applied for the recycling of magnets with non-ideal chemistries, containing impurity phases. This highlights the robustness of the process and its potential for use in short-loop recycling of Sm_2_TM_17_ sintered magnet production scrap. Crucially, this could offer a short-loop recycling avenue for Sm_2_TM_17_ production scrap that does not require recasting or the chemical recovery of alloying elements. Future work in this area will assess inert gas jet milling of HD powders generated from Sm_2_TM_17_ magnet production scrap and the blending of powder sources to compensate for ZrC inclusions.

## 5. Conclusions

This work investigated the hydrogen decrepitation (HD) of Sm_2_TM_17_ sintered magnet production scrap to assess its applicability as a recycling technique for compositions and/or microstructures that do not conform to commercial standards.

Production scrap samples were first investigated to examine irregularities in composition and magnetic properties compared to a commercial grade reference sample. The micro and nanostructure of the scrap was then analysed to investigate any impurity phases present that may have impacted magnetic performance. The scrap sintered magnets were then exposed to HD trials at 18 bar and 2 bar hydrogen pressure, at temperatures of 25–300 °C for 72 h, and the resultant material was characterised.

Initial characterisation of the scrap material showed that it had a demagnetisation trace with irregular domain wall pinning behaviour. This was attributed to ZrC inclusions which are non-magnetic and prevented cell structure formation locally. An abundance of grain boundaries within the scrap material, where the cellular structure was also shown not to precipitate, was also a potential reason for this discrepancy in magnetic properties.

HD trials conducted at 18 bar and 2 bar pressure yielded powder for applied temperatures of ≥50 °C. This thermal input was needed to satisfy the activation energy requirement for HD to occur. HD processing at 100 °C yielded high hydrogen contents of 0.299 Wt.% and 0.323 Wt.% for material reacted under 18 bar and 2 bar conditions respectively. This temperature also generated the greatest yield of powder, e.g., 75.6% < 1 mm for 2 bar trials.

Increasing the HD processing temperature above 100 °C to 300 °C showed a reduction in hydrogen uptake, with samples processed at 2 bar absorbing 68% less hydrogen at 300 °C. It is suggested that, much like with commercial samples, increased temperature restricted hydrogen loading by increasing the required pressure for optimal interstitial hydride formation. Additionally, reduced hydrogen absorption at greater temperatures was attributed to competing degassing reactions occurring at temperatures ≥ 150 °C. Increased processing temperatures led to coarser HD powder, whereas increased hydrogen pressure led to finer powder for equivalent temperature conditions.

Overall, this research suggests that HD processing of Sm_2_TM_17_ sintered magnet production scrap can be successfully undertaken using parameters similar to those previously applied to magnets of a commercial standard. This may allow for more energy-efficient short-loop recycling of production scrap material.

## Figures and Tables

**Table 1:** ICP-OES, oxygen, nitrogen and carbon analysis of Sm_2_TM_17_ production scrap compared against a Reference sample of correct production quality (normalised). Reference data adapted with permission from Ref. [15]. Copyright 2025 Elsevier, and production scrap data adapted from Ref. [23].

Sample	Sm	Co	Fe	Cu	Zr	O	N	C
	(Wt.%)	(Wt.%)	(Wt.%)	(Wt.%)	(Wt.%)	(ppm)	(ppm)	(ppm)
Production Scrap	23.8	49.1	19.3	4.7	2.8	1600	1100	870
	±0.16	±0.28	±0.10	±0.09	±0.12	±86	±84	±20
Reference Sample	24.6	48.7	18.9	4.7	2.9	2000	1100	130
	±0.12	±0.3	±0.1	±0.03	±0.15	±7	±11	±14

**Table 2:** (**A**) Collated magnetic properties of Sm_2_TM_17_ production scrap at operating temperatures of 25–200 °C. Reprinted from Ref. [23]. (**B**) Collated magnetic properties of Sm_2_TM_17_ commercial standard reference material at operating temperatures of 25–200 °C.

Temperature (°C)	Remanence, B_r_ (T)	Intrinsic Coercivity, H_cj_ (kA/m)	Maximum Energy Product, (BH)_max_ (kJm^−3^)
(**A**)			
25	1.21	>2055	>258
50	1.14	>1464	>232
100	1.13	1267	221
200	1.06	818	191
(**B**)			
25	1.16	>2158	>255
50	1.15	>1650	>245
100	1.11	>1648	>231
200	1.03	>1190	193

**Table 3:** EDS analysis showing the compositions of phases identified in the Sm_2_TM_17_ sintered magnet production scrap and the reference material. Production scrap data reprinted from Ref. [23].

EDS Site	Sm	Co	Fe	Cu	Zr
	(Wt.%)	(Wt.%)	(Wt.%)	(Wt.%)	(Wt.%)
Production Scrap Sample					
1	24.92 ± 0.41	48.82 ± 0.54	18.76 ± 0.31	5.30 ± 0.78	2.21 ± 0.17
2	56.41 ± 0.58	28.75 ± 0.41	10.59 ± 0.30	2.82 ± 0.71	1.44 ± 0.19
3	8.86 ± 0.36	15.16 ± 0.29	6.09 ± 0.22	1.24 ± 0.52	68.64 ± 0.54
Reference Sample					
1	25.41 ± 0.42	48.27 ± 0.54	19.24 ± 0.32	4.53 ± 0.81	2.56 ± 0.18
2	95.41 ± 0.27	4.59 ± 0.27	-	-	-

## Data Availability

The datasets presented in this article are not readily available due to the data being potentially commercially sensitive.
